# Supplementary material for: Analysis of Brain, Blood, and Testis Phenotypes Lacking the Vps13a Gene in C57BL/6N Mice
Source: Int J Mol Sci. 2024 Jul 16;25(14):7776. doi: 10.3390/ijms25147776 (PMC11277237; doi:10.3390/ijms25147776)
Supplement: Supplementary file 1 [file ijms-25-07776-s001.zip › ijms-3075664-Supplementary.pdf]

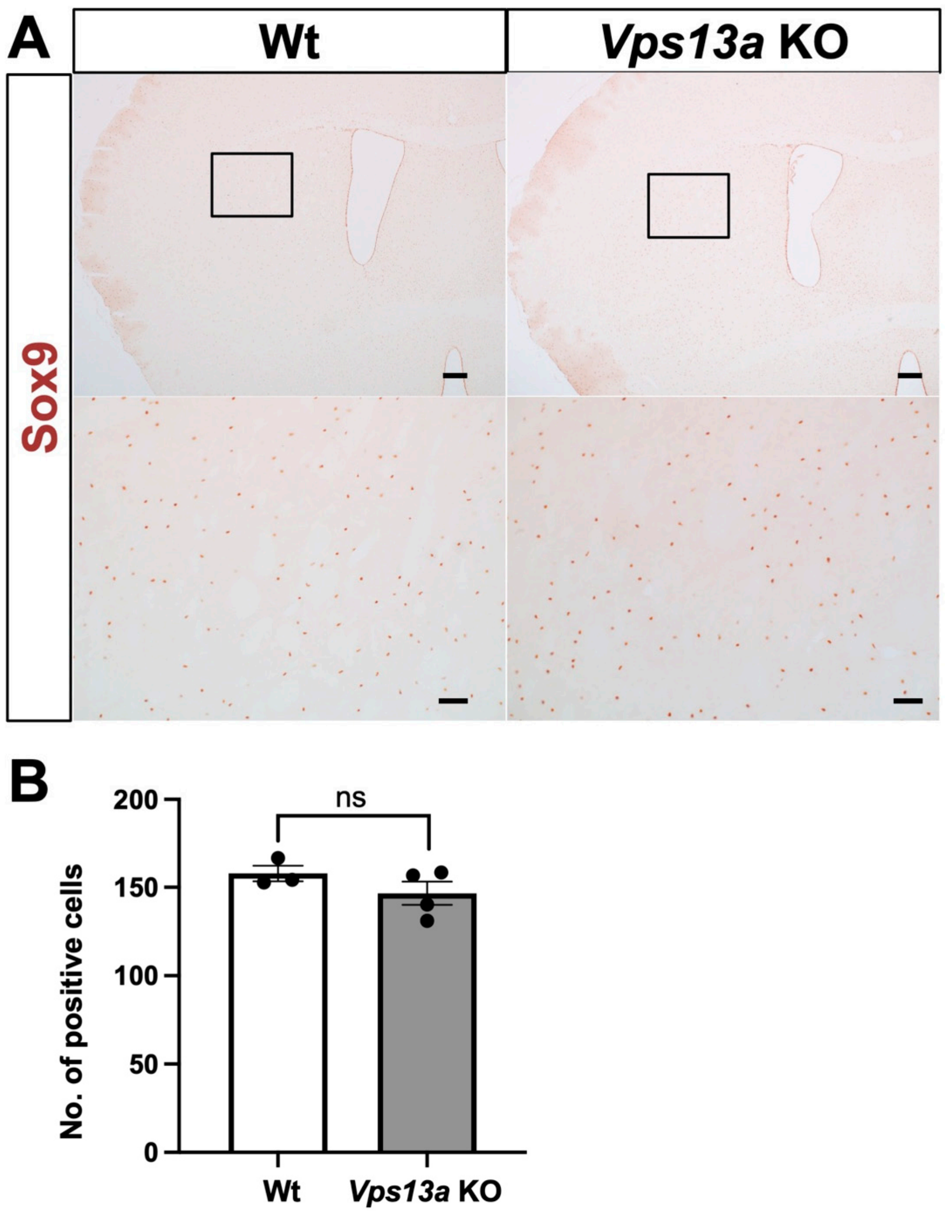

Supplemental Figure S1. Sox9 staining in the *Vps13a* KO brain.
(**A**) Sox9 IHC on coronal sections of Wt (left) and *Vps13a* KO brains (right) at 3 months old. (**B**) Quantification of Sox9-positive astrocytes in the Wt and *Vps13a* KO brains (n = 3-4 animals in each genotype). Scale bars: 200 µm (upper panel), 50 µm (lower panel). Results are shown as mean ± SEM, ns = not significant.

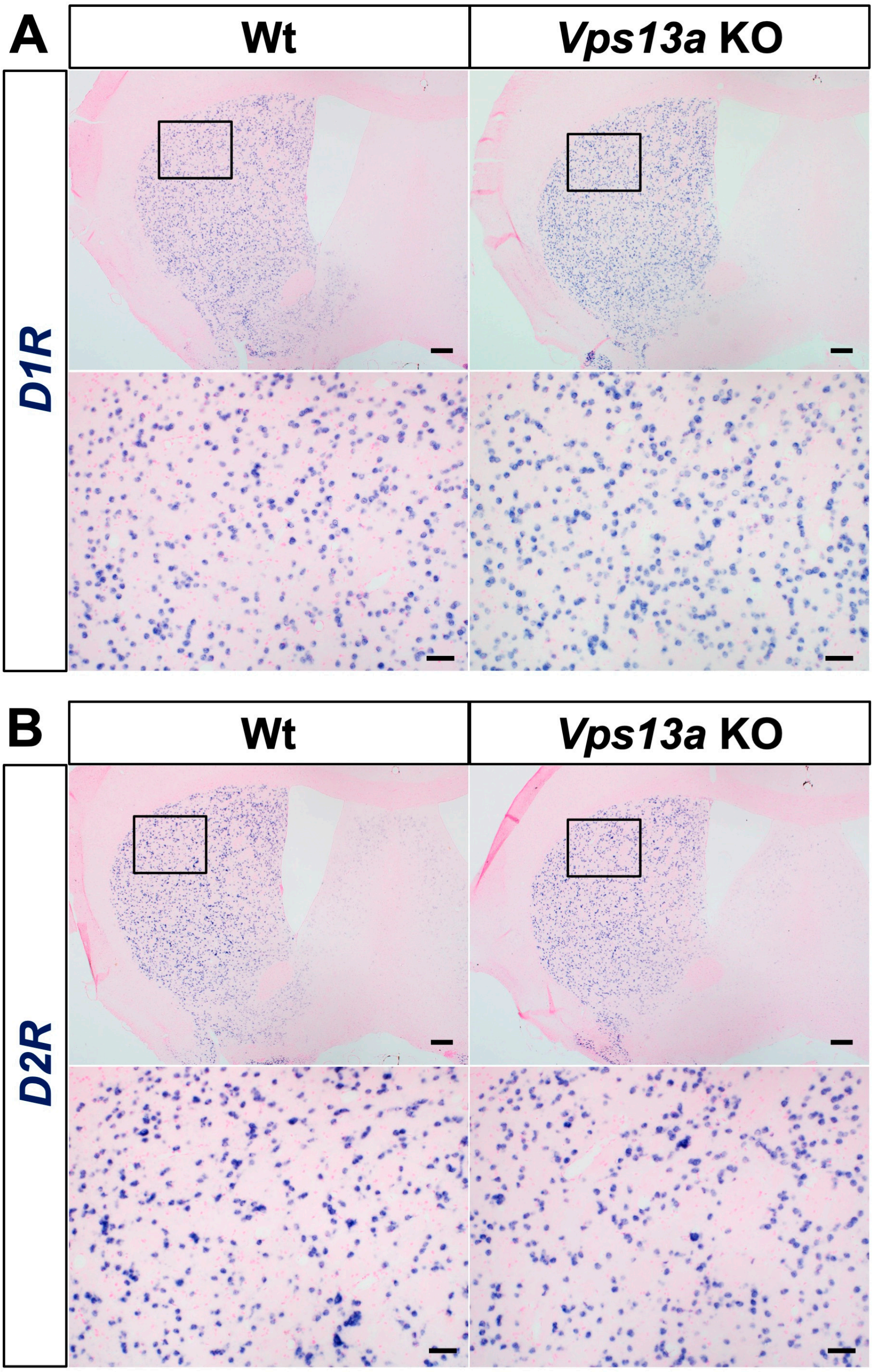

Supplemental Figure S2. ***D1R and D2R*** **mRNA expression in the forebrain at 18 months old**.
(**A**) *D1R* mRNA expression on coronal sections of Wt (left) and *Vps13a* KO (right) brains. (**B**) *D2R* mRNA expression on coronal sections of Wt (left) and *Vps13a* KO (right) brains ($n = 3$ animals in each genotype). Scale bars: 200 µm (upper panel), 50 µm (lower panel).

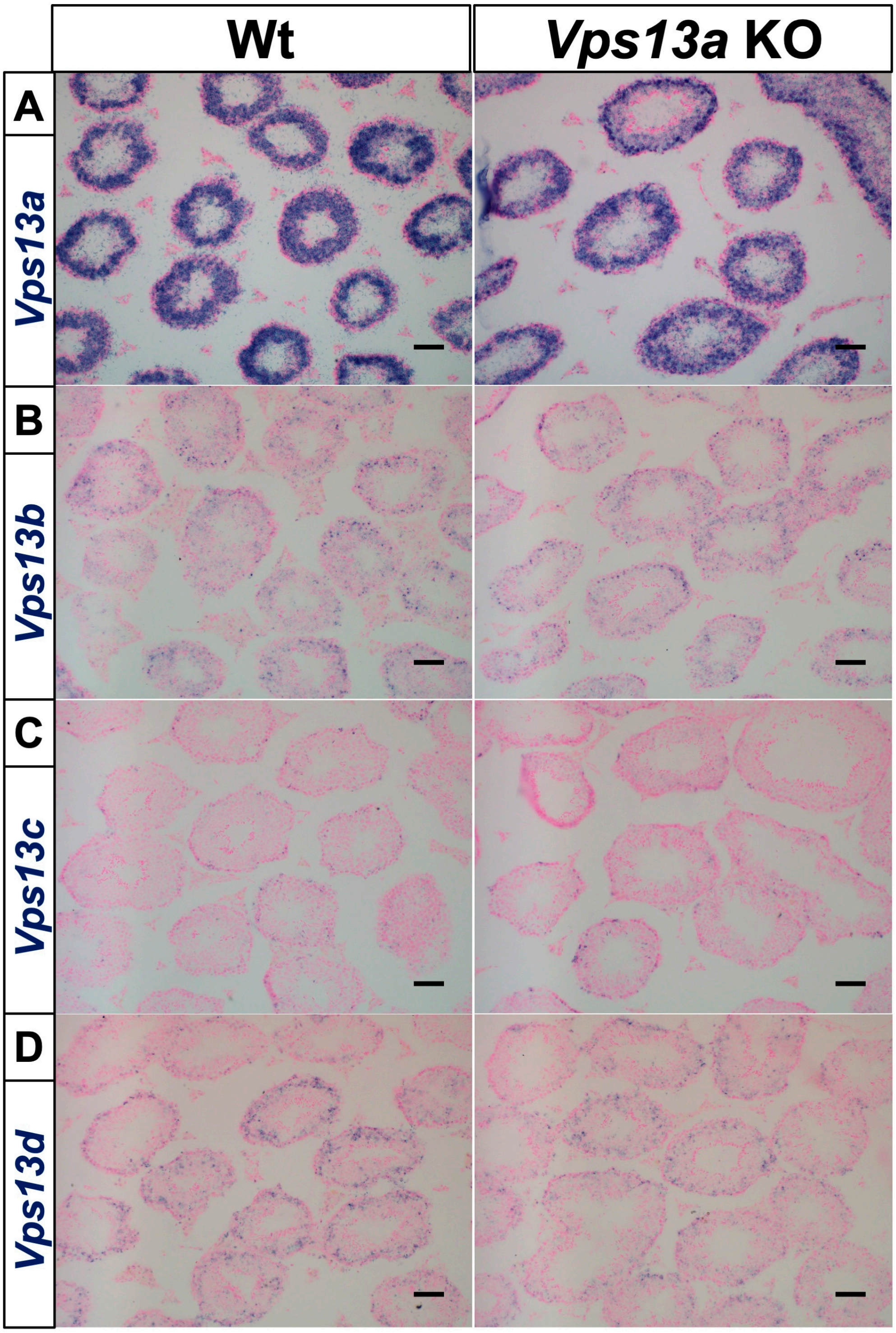

Supplemental Figure S3. ***Vps13a, Vps13b, Vps13c, Vps13d*** **mRNA expressions in the testis of** ***Vps13a*** **mutant mice.**

(**A**) *Vps13a* mRNA expression in the testis of Wt (left) and *Vps13a* KO (right). It is notable that this *Vps13a* probe detects transcripts from both Wt and null alleles. (**B**) *Vps13b* mRNA expression in the testis of Wt (left) and *Vps13a* KO (right). (**C**) *Vps13c* mRNA expression in the testis of Wt (left) and *Vps13a* KO (right). (**D**) *Vps13d* mRNA expression in the testis of Wt (left) and *Vps13a* KO (right). There were no big differences between Wt and *Vps13a* KO testis in all *Vps13* genes examined (n = 3 animals in each genotype). Scale bars: 50 μm.
